# Chemical cystitis due to crystal violet dye: a case report

**DOI:** 10.1186/1752-1947-7-145

**Published:** 2013-05-31

**Authors:** Takeshi Hashimoto, Makoto Ohori, Takeshi Kashima, Hidenobu Yamamoto, Masaaki Tachibana

**Affiliations:** 1 Department of Urology, Tokyo Medical University, 6-7-1 Nishishinjuku, Shinjuku-ku, Tokyo 1600023, Japan; 2 Department of Urology, Tokyo Metropolitan Hiroo Hospital, 2-34-10 Ebisu, Shibuya-ku, Tokyo 1500013, Japan

## Abstract

**Introduction:**

Crystal violet was commonly used for the treatment of oral and vaginal candidiasis or for sterilization during operations up to the 1960s. Because crystal violet is potentially toxic to mucosal membranes, it has been replaced with other disinfectants, and crystal violet is rarely used. We report a case of chemical cystitis due to intravesical instillation of crystal violet dye.

**Case presentation:**

Crystal violet dye was instilled into the bladder of a 47-year-old Japanese woman to confirm the presence of a vesicovaginal fistula. Our patient developed symptoms of gross hematuria, frequent urination and lower abdominal pain. Computed tomography showed thickening of her whole bladder wall with spotted high-density lesions. Cystoscopy demonstrated desquamated epithelial cells and a hemorrhagic bladder wall. We treated our patient conservatively with nonsteroidal anti-inflammatory drugs and glucocorticoids. During follow-up, magnetic resonance images showed that the detrusor muscle of her bladder was normal. Our patient’s symptoms gradually improved and she completely recovered within six months.

**Conclusion:**

Considering the severe side effect of crystal violet, it would be better not to use this dye to examine conditions such as a vesicovaginal fistula. Magnetic resonance imaging may help to evaluate the level of damage in the bladder wall of patients with chemical cystitis.

## Introduction

Crystal violet dye was commonly used for the treatment of oral and vaginal candidiasis or for sterilization during operations up to the 1960s [1-3]. Because crystal violet is potentially toxic to mucosal membranes, it has been replaced with other disinfectants, and crystal violet is rarely used. Crystal violet dye has also been used in the diagnosis of vesicovaginal fistula (VVF), including in Japan. It has now been replaced with indigo carmine solution. However, some older-generation gynecologists continue to use crystal violet for the diagnosis of VVF. We report a case of chemical cystitis due to crystal violet dye that was successfully treated with conservative therapy. This case should act to condemn the use of crystal violet.

## Case presentation

A 47-year-old Japanese woman, who was treated for cervical carcinoma with conization, returned to her gynecologist for a follow-up visit with a complaint of mucous hypersecretion and urinary leakage. Suspecting VVF, her gynecologist instilled 2ml of crystal violet dye diluted to 1% concentration. No VVF could be identified but the physician was unable to drain the dye from her bladder as his patient refused catheterization. She then developed an irritable bladder, frequent urination and gross hematuria. She was diagnosed to have acute cystitis and prescribed antimicrobial agents for one week. However, her symptoms did not improve and she was referred to our urology department.

**Figure 1:**
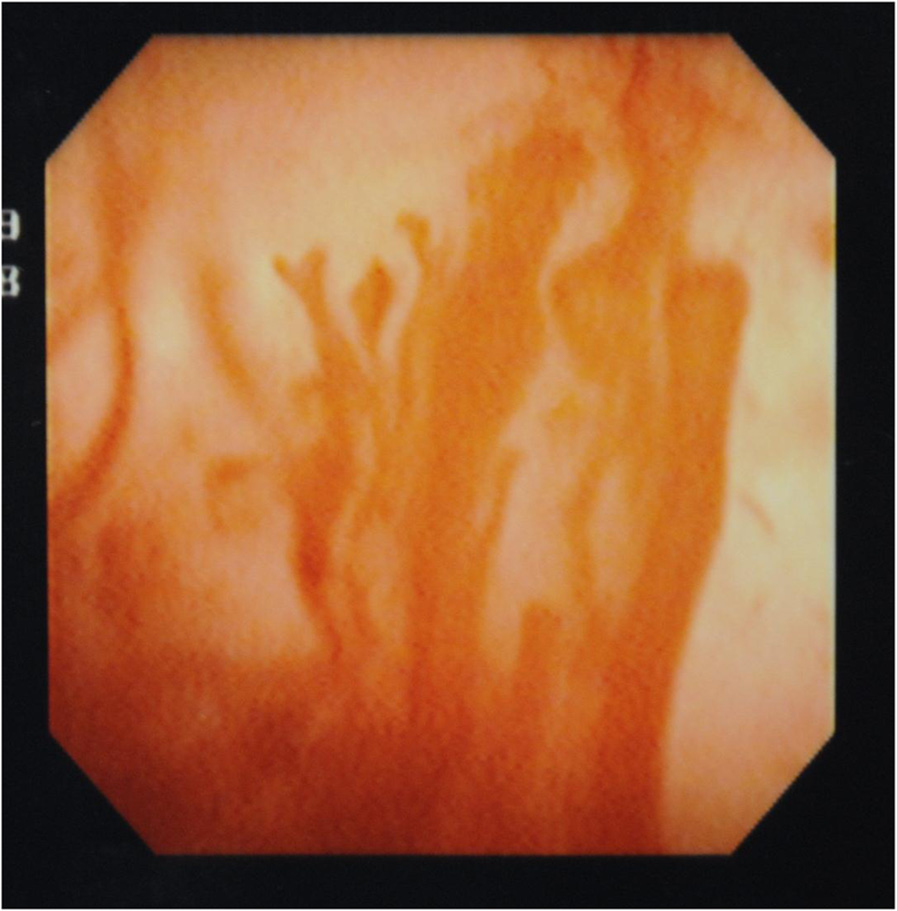
**The epithelial cells of the patient’s bladder wall had desquamated completely.** We could see profuse bleeding from the bladder surface.

A blood examination showed only a mild degree of elevated C-reactive protein. Her urinary sediment showed a red blood cell count of >100 cells/high-power field and a white blood cell count of >100 cells/high-power field. Her condition was gradually getting worse, with severe urgency to urinate every 10 minutes and gross hematuria with clots. Cystoscopy showed abnormal mucosa of her bladder. The surface of her bladder was hemorrhagic with a lot of necrotic tissue (Figure 1). Her bladder capacity was about 100mL.

**Figure 2:**
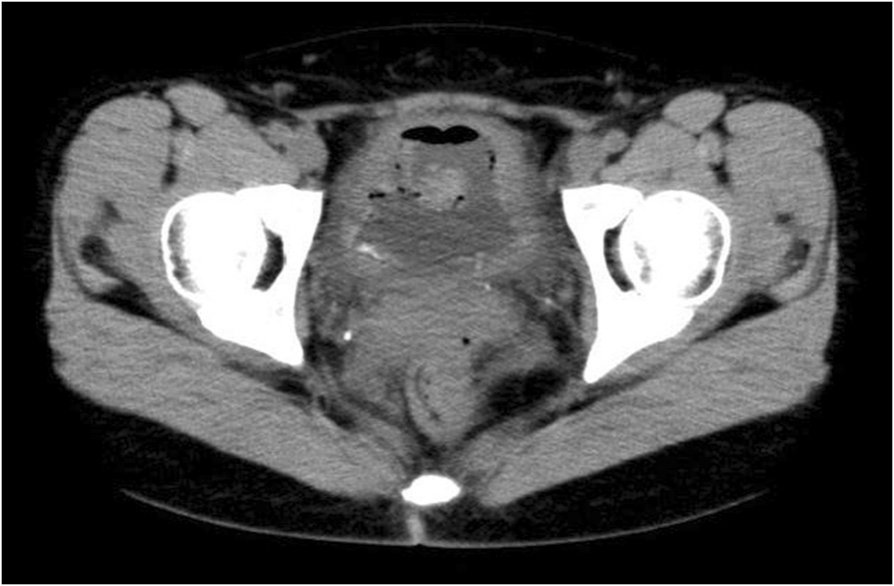
**Computed tomography imaging demonstrating thickening of the whole bladder wall and spotty high-density lesions of the bladder surface, with clotting blood.** A reduction in bladder capacity was suspected.

**Figure 3:**
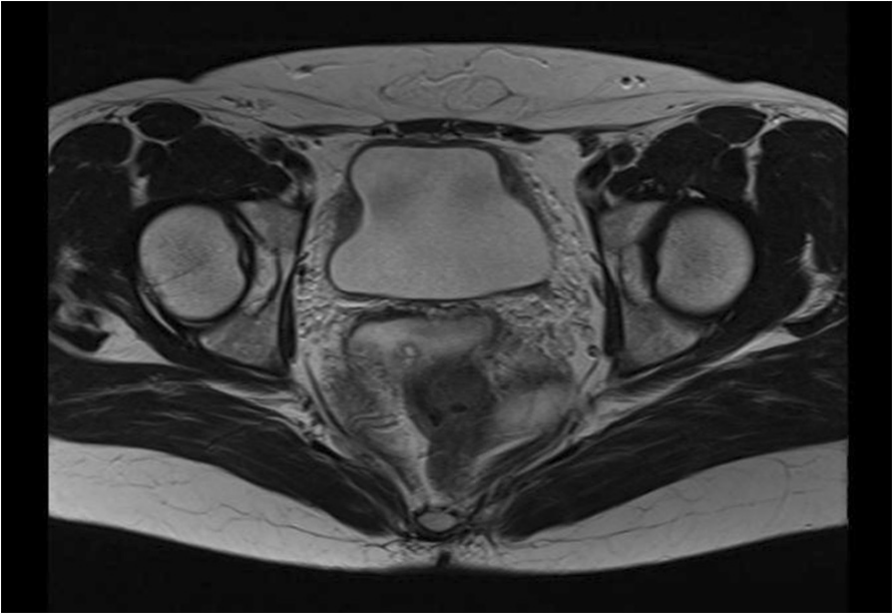
Magnetic resonance imaging after the bladder capacity improved to 75mL showed a well-rounded muscle layer.

We diagnosed our patient with chemical cystitis and started therapy with nonsteroid anti-inflammatory drugs (NSAIDs). An abdominal computed tomography scan showed thickening of her entire bladder wall with spotty high-density lesions of >100 Hounsfield units. We suspected these to be the dye, which had adsorbed into the mucosa (Figure 2). After two weeks, her urinary urgency had been relieved to some degree. However, her bladder capacity was only 50mL. We wanted to do a urodynamic study but our patient was not willing to undergo the procedure. We added glucocorticoids to her NSAID therapy to prevent scarring of her bladder as a result of inflammation. Her bladder capacity improved to 75mL, and pelvic magnetic resonance imaging (MRI) also showed an improvement in the thickening of her bladder wall and a normal muscle layer, which may indicate that the damage had not progressed to the muscle layer (Figure 3).

One month later, our patient had recovered from her severe symptoms. We changed the medication from a glucocorticoid to an anticholinergic agent. Two months after the change of medication, her bladder capacity had increased to 150mL, with a urination interval prolonged to 2 hours. Six months after our patient’s first visit, there were no obvious abnormalities in her bladder on cystoscopy or computed tomography.

## Discussion

Crystal violet, as well as gentian violet and methyl violet, belongs to a group of triphenylmethane dyes [4]. Similarly, Bonney’s blue also contains triphenylmethane dyes. Crystal violet has been used for the treatment of oral and vaginal candidiasis. In addition, it has been used to determine the patency of the fallopian tube and to demonstrate VVF [1]. However, since Slotkowski reported the case of a patient developing ulceration of oral mucosa as a result of treatment, crystal violet has been recognized as a potential irritant [5]. Crystal violet should therefore be diluted with water to a 0.5% concentration before intravesical instillation or contact with any other mucosal surface [1].

To the best of our knowledge, there have been three case reports of chemical cystitis due to intravesical instillation of crystal violet dye. The symptoms included gross hematuria, frequency of urination, urgent incontinence, enuresis, suprapubic discomfort and reduced bladder capacity. In two cases, chemical cystitis occurred after intravesical instillation of undiluted dye by mistake. A number of treatments such as hydraulic overdistention, augmentation ileocystoplasty, supratrigonal denervation, systemic steroids and intravesical treatment with dimethyl sulfoxide have been tried for this condition, with minimal benefit [2,6].

Kim *et al*. suggested that the degree of resulting damage depends upon the duration of exposure and the concentration of crystal violet [1]. Patients with instillation of diluted crystal violet at low levels of concentration had fewer symptoms and were able to recover from the symptoms with only conservative management. By contrast, it is sometimes very difficult to treat patients with an exposure of a long duration or high concentration [1,6].

Christmas *et al.* reported that the experimental instillation of 10% formaldehyde into rabbit bladders resulted in various degrees of cystitis according to the duration of exposure and the extent of the subsequent saline lavage. The damage produced ranged from superficial cystitis to transmural necrotizing cystitis [6]. The authors also commented that there is a dense submucosal neural plexus which is likely to be important in the perception of bladder sensation. The submucosal network is closely related anatomically to the urothelium lining the surface of the urinary bladder and is therefore potentially very susceptible to toxic damage by intravesical chemicals [6]. Once the bladder mucosa is damaged, subsequent neural damage and regeneration could lead to altered bladder sensation with associated alteration of the functional bladder capacity [7]. However, the nerves will recover as long as they are not entirely damaged.

A previous study described bladder changes on MRI in cases of radiation hemorrhagic cystitis. The patients presented with hematuria, and had increased bladder wall thickness on T1-weighted images and abnormal, high signal intensity in the outer bladder wall on T2-weighted images, secondary to chronic inflammation. MRI may provide important information on the extent of the damage [8]. In fact, in the present case, we were able to use MRI to determine that the damage caused by the crystal violet dye had been limited to the mucosa. Therefore, we believed that the injury to the submucosal neural plexus may have been partial and the subsequent reduction in bladder compliance and irritable bladder would be cured.

## Conclusion

Although chemical cystitis caused by crystal violet dye can be conservatively managed with NSAIDs and glucocorticoids, considering the severe side effects, it would be better not to use it to examine conditions such as VVF.

## Consent

Written informed consent was obtained from the patient for publication of this case report and any accompanying images. A copy of the written consent is available for review by the Editor-in-Chief of this journal.

## Abbreviations

NSAIDS: nonsteroidal anti-inflammatory drugs; MRI: magnetic resonance imaging; VVF: vesicovaginal fistula.

## Competing interests

The authors declare that they have no competing interests.

## Authors’ contributions

TH, TK and HY contributed to patient management. TH, MO and MT contributed to writing and reviewing the report. All authors read and approved the final manuscript.
